# Meta-Modeling of Methylprednisolone Effects on Glucose Regulation in Rats

**DOI:** 10.1371/journal.pone.0081679

**Published:** 2013-12-02

**Authors:** Jing Fang, Siddharth Sukumaran, Debra C. DuBois, Richard R. Almon, William J. Jusko

**Affiliations:** 1 Department of Pharmaceutical Sciences, School of Pharmacy and Pharmaceutical Sciences, State University of New York at Buffalo, Buffalo, New York, United States of America; 2 Department of Biological Sciences, University at Buffalo, State University of New York, Buffalo, New York, United States of America; Bascom Palmer Eye Institute, University of Miami School of Medicine, United States of America

## Abstract

A retrospective meta-modeling analysis was performed to integrate previously reported data of glucocorticoid (GC) effects on glucose regulation following a single intramuscular dose (50 mg/kg), single intravenous doses (10, 50 mg/kg), and intravenous infusions (0.1, 0.2, 0.3 and 0.4 mg/kg/h) of methylprednisolone (MPL) in normal and adrenalectomized (ADX) male Wistar rats. A mechanistic pharmacodynamic (PD) model was developed based on the receptor/gene/protein-mediated GC effects on glucose regulation. Three major target organs (liver, white adipose tissue and skeletal muscle) together with some selected intermediate controlling factors were designated as important regulators involved in the pathogenesis of GC-induced glucose dysregulation. Assessed were dynamic changes of food intake and systemic factors (plasma glucose, insulin, free fatty acids (FFA) and leptin) and tissue-specific biomarkers (cAMP, phosphoenolpyruvate carboxykinase (PEPCK) mRNA and enzyme activity, leptin mRNA, interleukin 6 receptor type 1 (IL6R1) mRNA and Insulin receptor substrate-1 (IRS-1) mRNA) after acute and chronic dosing with MPL along with the GC receptor (GR) dynamics in each target organ. Upon binding to GR in liver, MPL dosing caused increased glucose production by stimulating hepatic cAMP and PEPCK activity. In adipose tissue, the rise in leptin mRNA and plasma leptin caused reduction of food intake, the exogenous source of glucose input. Down-regulation of IRS-1 mRNA expression in skeletal muscle inhibited the stimulatory effect of insulin on glucose utilization further contributing to hyperglycemia. The nuclear drug-receptor complex served as the driving force for stimulation or inhibition of downstream target gene expression within different tissues. Incorporating information such as receptor dynamics, as well as the gene and protein induction, allowed us to describe the receptor-mediated effects of MPL on glucose regulation in each important tissue. This advanced mechanistic model provides unique insights into the contributions of major tissues and quantitative hypotheses for the multi-factor control of a complex metabolic system.

## Introduction

Due to their well-known anti-inflammatory and immunosuppressive properties, synthetic glucocorticoids (GC) are frequently prescribed for a broad spectrum of diseases such as allergic, inflammatory and autoimmune disorders. A study done in the United Kingdom reported that about 0.5% of the total population (65786 registered patients in 1995) received continuous (for at least three months) oral corticosteroid treatments [1]. However, many complications and undesirable effects are associated with chronic use of GC including muscle wasting, hyperglycemia, insulin resistance and/or diabetes mellitus. Multiple organs are involved in the pathogenesis of these disorders. In particular, GC-induced whole body insulin resistance is strongly related to the metabolic contributions of the most investigated tissues: liver, skeletal muscle and adipose tissue.

Many pathophysiological effects of GC are attributed to their transcriptional regulation of target genes. These effects are predominantly dependent on the interaction between GC and the glucocorticoid receptor (GR) [2]. As a ligand-activated transcription factor, the inactive GR in cytoplasm is bound to heat shock proteins (HSP), which prevents the nuclear localization of the receptor. After binding of specific GC ligands, the activated GR will dissociate from the HSP complex, undergo dimerization and nuclear translocation where it binds to GC response elements (GRE) in the promoter region of target genes. This process, together with other regulatory proteins, will enhance or repress the expression of these target genes. It is important to assess the role of GC on glucose regulation at systemic as well as gene levels.

Normal blood glucose concentrations are sustained mainly through the balance between hepatic glucose output (HGO) and glucose uptake primarily by brain, muscle and adipose tissue. Liver is a primary metabolic target of GC. The major sources of HGO are from glycogenolysis and gluconeogenesis. The GC have a major influence on gluconeogenesis by affecting the availability of gluconeogenic precursors and the activity of several key gluconeogenic enzymes including phosphoenolpyruvate carboxykinase (PEPCK) [3]. The GC increase the expression and activity of PEPCK, thereby augmenting gluconeogenesis and increasing HGO. In addition, cAMP in liver also plays an important role in stimulating glucose production, either by enhancing PEPCK activity or by stimulating glycogen breakdown [4]. Furthermore, GC stimulate protein breakdown and the release of amino acids from skeletal muscle, as well as increase lipolysis and mobilization of glycerol and fatty acids from adipose tissue [5]. Amino acid carbon from skeletal muscle and glycerol from adipose tissue provide substrates for hepatic gluconeogenesis.

White adipose tissue is a complex metabolic and endocrine organ that is also an important GC target [6]. In addition to its role as the reservoir of excess energy, the adipocytes also release hormones, adipokines, to communicate with other organ systems. The GC stimulate lipolysis, resulting in increased plasma FFA concentrations [7], [8]. The FFA serve as an energy source through beta-oxidation and diminish glucose uptake and utilization by skeletal muscle. Literature data suggest that FFA promote insulin secretion upon acute exposure, however long-term oversupply of FFA leads to impaired pancreatic insulin secretion [7], [9]. Besides FFA, some adipocyte-derived proteins have been shown to regulate insulin sensitivity including leptin and adiponectin. Leptin expression is regulated by GC both *in vitro* and *in viv*o [10], [11], [12], [13]. Rats had increased plasma leptin concentrations after acute and chronic dosing of GC in a dose-dependent manner [10]. Leptin suppresses feeding and regulates energy expenditure and hence contributes to systemic energy homeostasis [14], [15]. In addition, leptin exerts its effects by interacting with specific leptin receptors in the central nervous system, thus altering downstream functional mediators [16], [17].

Skeletal muscle is an important target for GC-induced insulin resistance. Effects of GC on muscle carbohydrate and protein metabolism are of importance owing to the fact that muscle accounts for about 80% of insulin-stimulated glucose disposal and represents a major source of gluconeogenic precursors. Dosing with GC reduces insulin-stimulated glucose uptake and increases proteolysis in skeletal muscle [18]. In addition, our microarray studies have shown a significant decrease in muscle IRS-1 mRNA expression following acute and chronic MPL in ADX rats [19], which is in agreement with decreased muscle IRS-1 protein concentrations after chronic GC use [20]. The IRS-1 transmits signals from insulin to intracellular signaling pathways and plays a key role in downstream gene regulation. As a central player in insulin signaling pathways, the decline in IRS-1 will reduce insulin-dependent glucose uptake in muscle, which may in part explain the impaired peripheral glucose utilization after GC dosing.

Based on the accumulated clinical evidence linking GC in the pathogenesis of diabetes, extensive studies have been conducted by our laboratory to examine the mechanisms by which GC alter glucose metabolism and induce insulin resistance in rats [21], [22], [23]. The effects of acute and chronic MPL on glucose homeostasis were investigated [4], [24], [25], [26], [27] and a series of mechanistic PK/PD models were proposed to explain the time course of systemic glucose changes. The important role of white adipose tissue was appreciated [24]. However, as GC-induced glucose dysregulation is a whole body metabolic syndrome, the interplay of multiple target organs was not assessed simultaneously. Multiple intermediary controlling factors may mediate GC action and glucose regulation.

Therefore, a more “complete” mechanistic systems model was sought to include essential biomarkers from multiple tissues. Parallel analysis and measurements of multiple tissue-relevant factors offers better understanding of drug action and pathophysiology, and helps identify more relevant controlling factors. This model includes receptor binding components and target gene mRNA in different organs to more fully account for the receptor/gene-mediated GC effects. Our fifth-generation receptor/gene-mediated GC PD model has been applied to numerous data sets [3], [28]. The effects of MPL on the dynamics of GR mRNA, free cytosolic receptor, and drug-receptor complex as studied in several target tissues [4], [19], [24] were incorporated into this systems model. Due to the complexity in model fitting, piecewise fitting techniques were applied with initially estimated parameters fixed for subsequent data analysis. Previous knowledge and experimental findings from short- and long-term dosing effects of MPL on glucose regulation were integrated in a more generalized mathematical meta-model.

The aim of quantitative and systems pharmacology is to expedite the drug discovery process and to understand mechanisms of drug actions by developing mathematical models. There are two general approaches to modeling experimental data in quantitative pharmacology. The traditional pharmacometrics approach involves developing a quasi-mechanistic model and using fitting procedures to capture model parameters and sources of variability. Fundamental principles of target occupancy, turnover and signal transduction are often incorporated into the model building processes in mechanistic terms [29]. A trial-and-error process is involved with employment of various fitting justifications and qualification criteria. This is considered as a “top-down” approach. The other is a “bottom-up” approach taken by systems biologists to assemble complex biochemical and physiological schemes, and synthesize the acquired information into a large mathematical model [30]. Model parameter values are usually assigned based on judgments of best information from the literature. Simulations are then made to test model predictions against experimental data. It was pointed out at the first NIH-sponsored meeting in 2008 on ‘Quantitative and Systems Pharmacology’ that opportunities and challenges exist for developing small systems models using yet-to-be-devised intermediary methods between model-fitting and simulations (http://www.nigms.nih.gov/News/Reports/201110-syspharma.htm). This report provides such a meta-modeling effort where data and models from multiple studies are melded to determine how well an array of hypotheses on how corticosteroids alter glucose metabolism helps explain the overall experimental data.

## Materials and Methods

### Animals

This study involves five rat experiments performed in our laboratory (Table 1). Extensive descriptions were published [3], [24], [25], [26], [27]. Briefly, all animal experiments consisted of male Wistar rats that were acclimatized for at least 1 week to a 12 h/12 h light-dark cycle. Rats were housed at constant temperature (22°C) with free access to water (intact rats) or saline (adrenalectomized rats) along with standard rat chow. The research adhered to the ‘Principles of Laboratory Animal Care’ and was approved by the State University of New York at Buffalo Institutional Animal Care and Use Committee. In the chronic infusion study [25], 32 normal Wistar rats were randomly divided into 6 sub-groups which received saline or MPL infusions at the rate of 0.03, 0.1, 0.2, 0.3, or 0.4 mg/kg/h via Alzet osmotic mini-pumps (Model 2ML4, flow-rate 2.5 µl/h, Alza Corp., Palo Alto, CA). Rats were sacrificed at various times over 21 days for low dose groups (0.03 and 0.1 mg/kg/h), over 10 days for the medium dose group (0.2 mg/kg/h), and 7 days for high dose groups (0.3 and 0.4 mg/kg/h). In the circadian-nadir study [24], 54 normal Wistar rats received 50 mg/kg MPL IM at the nadir of the corticosterone rhythm (1.5–3.5 h after lights on) and sacrificed at various circadian time points after drug dosing. In the single IV injection and short-term infusion studies [3], [26], [27], two different groups of rats (normal and ADX) were given MPL IV at 10 or 50 mg/kg, or 7-day continuous infusions at the rate of 0.1 or 0.3 mg/kg/h via Alzet osmotic mini-pumps (Model 2001, flow-rate 1.0 µl/h, Alza Corp.). In all studies, control rats were also sacrificed at various time points.

**Table 1:** Experimental designs and observations from different studies.

Experiments	Measurements	Not measured
Injection and short infusion studies		
	Glucose	Food intake
_Jin JY et al. _ [26], [27]	Insulin	Leptin
_Yao ZL et al. _ [_19_]	cAMP, PEPCK mRNA, PEPCK, GR dynamics	Glucose, Insulin, Leptin
	IL6R1 mRNA and IRS-1 mRNA	
Circadian-nadir		
	Glucose GR mRNA in adipose tissue	Food intake
Sukumaran et al. [24]	Insulin PEPCK mRNA loss rate constant	
	FFA Leptin Stimulation of PEPCK mRNA production	
	Leptin mRNA, Leptin	
	GR mRNA in adipose tissue	
Chronic infusion		
	Glucose GR mRNA in adipose tissue	GR dynamics
Fang et al. [25]	Insulin PEPCK mRNA loss rate constant	Leptin mRNA
	FFA Leptin Stimulation of PEPCK mRNA production	cAMP, PEPCK dynamics
	Leptin	
	Food intake	

### Assays

Plasma MPL and corticosterone concentrations were determined by a normal-phase high-performance liquid chromatography (HPLC) as previously described [31]. In the chronic infusion study [25], plasma insulin was measured using the Rat/Mouse Insulin ELISA kit (Millipore Corporation, Billerica, MA). Blood glucose was measured using a BD Logic Blood Glucose Monitor (BD, Franklin Lakes, NJ). The recommended blood/plasma conversion: Plasma Glucose = 1.11 · Blood Glucose [32] was used. Plasma free fatty acids (FFA) were measured using a commercial enzymatic colorimetric assay (Roche Applied Sciences, Indianapolis, IN) adapted to a 96-well plate format with standard curves prepared from a commercial standard solution (WAKO NEFA; Wako Pure Chemicals, Richmond, VA). Plasma leptin was measured at several time points in control, low dose (0.03 mg/kg/h) and high dose (0.3 and 0.4 mg/kg/h) groups by commercial enzyme-linked immunosorbent assay (Rat Leptin EIA; Assay Designs, Ann Arbor, MI). In the circadian-nadir study [24], plasma insulin was measured using a commercial radioimmunoassay (RI-13K Rat Insulin RIA Kit; Millipore Corporation). Plasma glucose was measured by the modified glucose oxidase method (Sigma GAGO-20; Sigma-Aldrich, St. Louis, MO). Plasma FFA was quantified using the nonesterified fatty acids detection kit (Zen-Bio, Research Triangle Park, NC) and standard curves were constructed from a commercial standard (WAKO NEFA). Plasma leptin was measured by commercial enzyme-linked immunosorbent assay (Rat Leptin TiterZyme EIA; Assay Designs, Ann Arbor, MI). In the injection and short-term infusion studies [3], [26], [27], plasma insulin was measured by a rat-specific enzyme-linked immunosorbent assay (1-2-3 Rat Insulin ELISA, ALPCO Diagnostics, Windham, NH). A modified glucose oxidase method (Sigma Diagnostics) was used to determine plasma glucose. Hepatic cAMP, PEPCK mRNA and hepatic PEPCK activity were analyzed by real-time qRT-PCR with methodology described previously [3].

### Quantitative Real-Time Reverse Transcription-Polymerase Chain Reaction Measurements

Hepatic cytosolic free glucocorticoid receptor (GR) density and mRNA data were quantified as described previously [28]. Abdominal fat GR and leptin mRNA were determined by real-time qRT-PCR using TaqMan-based probes in the circadian-nadir study [24]. The GR density and its mRNA in gastrocnemius muscles were also measured previously [33].

### Pharmacokinetic/Pharmacodynamic Model

#### Pharmacokinetics

For IV injection and infusion studies [25], [26], [27], the PK of MPL was described by a two-compartment model with appropriate input functions [26]. For the IM study, two absorption pathways from the injection site were added [34]. The PK symbols are listed in Table 2. The PK parameters were fixed from our previous studies [26], [34] and plasma drug concentrations (*C_MPL_*) were used as the driving force in the following dynamic analysis.

**Table 2:** Pharmacokinetic parameters of methylprednisolone.

Parameter (units)	Definition	Value	CV%
*CL* (L/h/kg)	Clearance	4.91^a^	10
*V_p_* (L/kg)	Central volume	1.17a,b	50
*k_12_* (h^−1^)	Distribution rate constant	0.39a,b	10
*k_21_* (h^−1^)	Distribution rate constant	0.78a,b	20
*k_el_* (h^−1^)	Elimination rate constant	5.57***^b^***	27
*F*	Bioavailability (IM)	0.214***^b^***	16
*F_r_*	Fraction absorbed by k_a1_ (IM)	0.725***^b^***	11
*k_a1_* (h^−1^)	Absorption rate constant	1.255***^b^***	23
*k_a2_* (h^−1^)	Absorption rate constant	0.219***^b^***	54

a IV injection; ***^b^*** IM injection. Parameter values were obtained from Jin et al. [26] and Hazra et al. [37]

#### Pharmacodynamics

**Figure 1:**
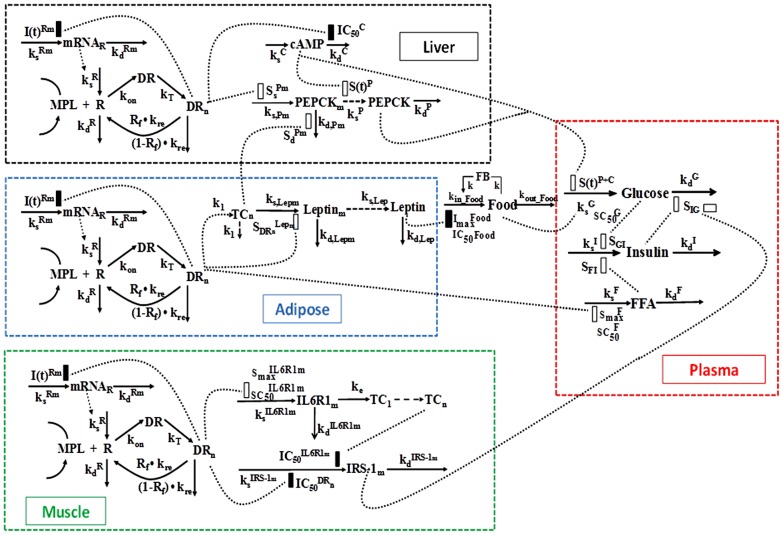
Schematic representation of the receptor/gene/protein mediated PD model of MPL effects on glucose regulation with different colored boxes indicating major target tissue-associated events (black: liver; blue: white adipose tissue; green: skeletal muscle; and red: plasma). Differential equations for the model are defined in Eq. (5) – (24). The dotted lines and rectangles indicate inhibition (closed bar) and stimulation (open bar) of the various processes. Symbols are defined in the text and tables.

The extended glucose regulation model for receptor/gene/protein mediated GC effects is shown in Fig 1. The GC effects in different tissues [4], [24], [33] are incorporated and adaptations were applied to the effects of GC on selected hepatic, white adipose tissue and skeletal muscle biomarkers. In brief, the current systems model was constructed based on previous study results and models developed in our lab: the fifth-generation receptor/gene mediated GC PD model has been applied to describe the effects of MPL on the dynamics of GR mRNA, free cytosolic receptor and nuclear drug-receptor complex in liver, skeletal muscle and white adipose tissue [4], [24], [33]. For each of these target tissues, the mechanisms underlying GC receptor dynamics are assumed to be similar. The nuclear drug-receptor complex served as the driving force to regulate downstream target gene/protein expression in relation to glycemic control. Upon acute and/or chronic dosing of MPL, receptor/gene-mediated GC responses were assessed previously in our lab including hepatic cAMP, PEPCK mRNA and hepatic PEPCK activity [3], IL6R1 and IRS-1 mRNA in skeletal muscle [19], adipocyte-derived cytokines such as leptin mRNA and plasma leptin [24], and systemic biomarkers such as plasma glucose, insulin, and FFA as well as food intake [4], [24], [25]. Therefore, previously generated experimental data and modeling results provide a basis to inform and test our current systems model. The basic glucose-insulin feedback model was selected [4] and extended by including the FFA component with multiple physiological interactions with glucose and insulin [24], [25]. Leptin and Food intake influences were incorporated to characterize the effects of exogenous nutrient supply [25]. Various PD markers aforementioned in those three target tissues were tested and included in our current model. Partitioning the systems into different sub-models is described in detail in the following sections.

### Glucocorticoid Receptor Dynamics

The dynamics of GR were previously studied in liver, muscle and white adipose tissue [4], [24], [33], [34], [35]. The receptor dynamic parameters governing drug-receptor binding, translocation and recycling obtained previously [4], [24], [33], [34] were fixed. The GC receptor/gene mechanisms are considered to be similar in different tissues. Therefore, the differential equations and initial conditions (IC) for GC receptor dynamics are the same for all tissues as follows:
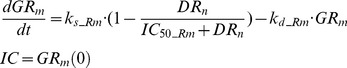
(1)

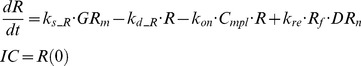
(2)

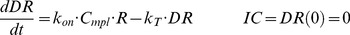
(3)

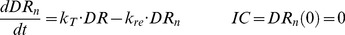
(4) where symbols are the mRNA for GR (*GR_m_*), free cytosolic receptor density (*R*), and cytosolic (*DR*) and nuclear (*DR_n_*) drug-receptor complex concentrations. Symbols *k_s_Rm_* and *k_d_Rm_* are zero-order synthesis rate and first-order degradation rate constants for GR mRNA, *k_s_R_* and *k_d_R_* are first-order synthesis and degradation rate constants for free receptor (*R*). Other symbols include: the drug-receptor second-order association rate constant (*k_on_*), DR nuclear translocation and recycling rate constants (*k_T_* and *k_re_*), fraction of recycling receptors (*R_f_*), and the concentration of DR_n_ at which the synthesis rate of GR_m_ is reduced to 50% of its baseline (*IC_50_Rm_*).

### Leptin Dynamics and Food Intake

An indirect response model was used previously to describe the reduction of daily food consumption (*Food*, kilocalories per day) during MPL infusions [25], assuming an inhibitory effect of plasma MPL on the input rate for food (*k_in_Food_*). Keeping the basic structure model for food intake, we extended the model by incorporating the receptor dynamics, leptin mRNA, and plasma leptin information from the circadian-nadir study [24] and measurements of plasma leptin during MPL infusions. The updated model for effects of MPL on food intake is depicted in Fig. 1. Parameters from modeling the leptin data from two studies [24], [25] without incorporating circadian oscillations were obtained and fixed as the driving force in the PD analysis of food intake. The stimulatory effect of MPL on the production rate (*k_s,Lepm_*) of leptin mRNA by the activated nuclear drug-receptor complex (*DR_n_*) assumes a stimulatory constant 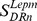. The symbol 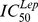 is the plasma leptin concentration causing 50% inhibition of input rate of food. The equations and IC are:
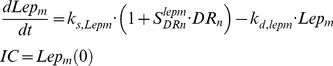
(5)

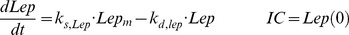
(6)

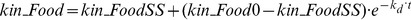
(7)

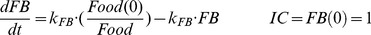
(8)

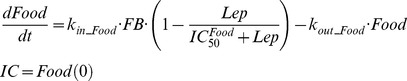
(9)


The stress effect on food intake and a feedback step (*FB*) were modeled as previously [25] where the input rate of food (*k_in_Food_*) was described by an exponential decline from *k_in_Food0_* to a new steady-state *k_in_FoodSS_*. The *k*_in_Food0_ = *k*_out_Food_ • *Food*_max_, where *Food*_max_ indicates hypothetical maximal food consumption at steady-state [25]. A first-order rate constant *k_d_* was used to capture the delayed tolerance effect on *k_in_Food_*. Similarly, we used indirect response model I (inhibition of input) to model the inhibitory effect of leptin on the input rate of food intake *k_in_Food_*.

### Cyclic AMP and PEPCK Dynamics

Detailed descriptions of the PD models used to describe hepatic cAMP, PEPCK mRNA and PEPCK activity after MPL dosing are available [3]. The receptor/gene/protein-mediated GC effects on glucose regulation and selected liver biomarkers are depicted in Fig.1. The dynamics of hepatic cAMP concentration (*cAMP*), hypothetical biosignal (*TC*) produced by *DR_n_*, hepatic PEPCK mRNA concentration (*PEPCK_m_*), and PEPCK activity (*PEPCK*) are described by:
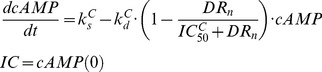
(10)

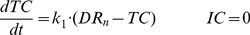
(11)

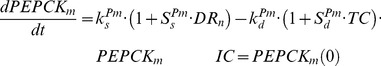
(12)

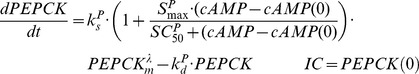
(13)where 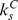 and 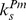
are the zero-order synthesis rates of cAMP and PEPCK mRNA. Other rate constants include: the first-order degradation of cAMP (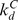), PEPCK mRNA (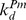), and PEPCK (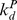), the first-order synthesis rate of PEPCK (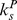), and TC transduction (*k_1_*). The 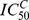 is the concentration of *DR_n_* required to inhibit cAMP degradation by 50%. The 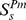
and 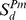
are linear efficiency factors for *DR_n_* and *TC* stimulating PEPCK mRNA production and degradation. The 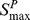
and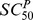 describe the maximum stimulation of PEPCK synthesis by the change of cAMP and the increase of cAMP needed to produce half maximal stimulation. The *λ* is the amplification factor to account for the translation from PEPCK mRNA to PEPCK protein. The PD parameter values for cAMP and PEPCK dynamics were fixed according to our previous report [3]. The time course changes of cAMP and PEPCK were simulated after various dosing regimens of MPL.

### IRS-1_m_ Dynamics

The effect of MPL on the mRNA expression of IRS-1 (*IRS1_m_*) in rat skeletal muscle was modeled previously [19]. In addition to the direct inhibitory effect of nuclear drug-receptor complex (*DR_n_*) on the transcription of IRS-1, the model incorporated an intermediate controller of IL-6 receptor type 1 mRNA (*IL6R1_m_*) which exerts long-term inhibitory effects on the production of IRS-1 mRNA. A series of transit compartments (*TC_n_*) with a first-order rate constant (*k_e_*) were needed to explain the delayed effect of IL6R1_m_ on the synthesis rate (*k_s_m_*) of IRS1_m_. The model equations are:
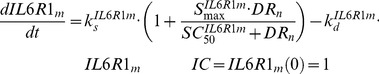
(14)

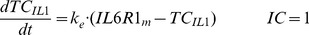
(15)

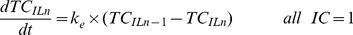
(16)

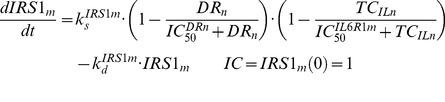
(17)where 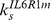 and 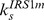
are zero-order synthesis rates of IL6R1 and IRS-1 mRNA; 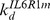 and 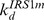
are first-order degradation rate constants for IL6R1 and IRS-1 mRNA; the 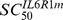
is the *DR_n_* concentration required to exert 50% of maximum stimulation (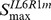) of IL6R1 mRNA transcription; 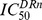 is the *DR_n_* concentration at which the synthesis rate of *IRS1_m_* is reduced by 50%, and 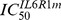
reflects the *TC_n_* value that reduces the synthesis rate of *IRS1_m_* by 50%. The PD parameters for *IL6R1_m_* and *IRS1_m_* dynamics were fixed as reported [19].

### Glucose-Insulin-FFA Dynamics

Based on mechanistic reasons, our PD model was developed incorporating GC stimulation of FFA production via receptor-mediated effects in white adipose tissue, the effects of hepatic PEPCK and cAMP on glucose production [4], glucose-insulin-FFA inter-regulation including glucose/FFA stimulation of insulin secretion, insulin stimulation of glucose/FFA disposition, and the negative feedback of *IRS1_m_* on insulin stimulation of glucose utilization (Fig. 1). The effect of exogenous glucose input from food intake utilized a linear efficiency constant *S_Food_* acting on the rate of glucose synthesis [25]. In the circadian-nadir study [24], different rats were sacrificed at each time point and food intake was not monitored. Hence the parameter 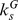
was a combined indicator for both exogenous and endogenous glucose production. The basic structure of the glucose-insulin-FFA feedback system was described previously [25]. The following equations were used to fit glucose (*G*), insulin (*I*) and FFA (*FFA*) data from all MPL doses simultaneously:
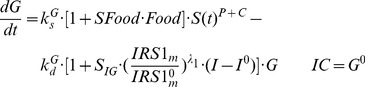
(18)

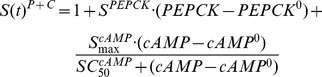
(19)

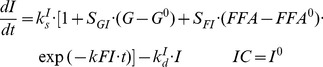
(20)

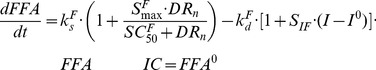
(21)


Glucose turnover has production with a zero-order rate constant 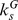
and utilization with a first-order rate constant 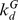. The PEPCK and cAMP increase glucose production presumably by stimulating gluconeogenesis and glycogenolysis in liver. The *S^PEPCK^* is the linear efficiency factor of PEPCK stimulation of glucose production. The 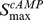
and 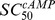
reflect the nonlinear stimulation of 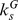 by changes in cAMP. Similar to previous models [25], the increase of insulin from baseline (*I^0^*) regulates glucose by stimulating its disposition with a linear efficiency constant *S_IG_*. The ratio of *IRS1_m_*/*IRS1_m_^0^* together with a power coefficient *λ_1_* reflect the impairing effect of IRS-1 mRNA on insulin stimulation of glucose utilization [26].

In Eq. (20), insulin turnover is described by a zero-order production process 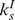 and first-order degradation rate constant 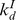. The change of glucose from baseline (*G^0^*) is used to drive the stimulation effect of glucose on insulin production with a linear efficiency constant *S_GI_*. In the chronic infusion study, the effect of FFA on insulin is incorporated as previously described [25] with an empirical function *exp(-k_FI_·t)* modulating the time-dependent stimulation effect of FFA on insulin secretion. In the circadian-nadir and IV injection studies, the acute effects of FFA on insulin production was modeled with a linear efficiency constant *S_FI_* and the change of FFA from baseline (*FFA^0^*). In Eq. (21), FFA is constantly produced at a zero-order rate constant 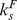 and utilized with a first-order rate constant 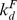. The stimulatory effect of MPL on FFA production is presumed via the activated nuclear drug-receptor complex (*DR_n_*) in white adipose tissue with a maximum achievable stimulation constant 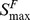 and the sensitivity parameter 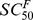. The change of insulin from baseline (*I^0^*) is used to control FFA disposition with a linear efficiency constant (*S_IF_*).

The system was assumed to be at steady-state at time zero, and Eq. 18, 20, 21 yield:
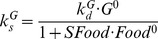
(22)

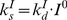
(23)

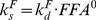
(24)where initial values of *G^0^*, *I^0^*, and *FFA^0^* were fixed as the mean baseline values for each dose group.

### Data Analysis

Data taken from five animal studies were pooled for simultaneous modeling [3], [24], [25], [26], [27]. Table 1 gives the summary of measurements that were made in these studies. Due to the complexity in model fitting, a piecewise approach was applied for fitting the plasma glucose, insulin and FFA data. With this approach the reported PK parameter values were fixed to serve as a driving force for the PD effects after various MPL dosing regimens [26], [37]. GR dynamic parameters in liver and muscle were fixed based on previously reported results [3], [19] to permit usage of time-course changes of nuclear drug-receptor complex (DR_n_) as an input function for regulation of downstream target gene/protein expression in each tissue. Additionally, MPL effects on IL-6R1, IRS-1 in muscle, and cAMP/PEPCK dynamics in liver were fixed with previously reported values [3], [19]. Further, sensitivity analysis for parameters describing GR dynamics in liver and muscle were performed using the Berkeley Madonna program (University of California at Berkeley, CA). In adipose tissue, GR dynamics was first estimated separately utilizing the mRNA expression data and the parameters were fixed for the subsequent step in modeling the effects of drug dosing on leptin mRNA and protein expression in adipose tissue. These estimated parameters were further fixed for modeling the effects of leptin on food intake. As the final step, previously resolved PD parameters governing MPL effects on PEPCK, cAMP in liver, IRS-1 in muscle, food intake and DR_n_ in adipose tissue were fixed to provide time-course input functions, which permit further estimations of some of the key PD parameters linking those intermittent biomarkers to the systemic changes of plasma glucose/FFA/insulin.

The ADAPT 5 software [36] was used for all model fittings with the maximum likelihood method. The variance model was defined as *V(σ,θ,t_i_)* = *σ_1_^2^• Y(θ,t_i_)^σ2^*, where *V(σ,θ,t_i_)* is the variance for the ith point, *Y(θ,t_i_)* is the ith model-predicted value, *θ* represents the estimated structural parameters, and *σ_1_*, *σ_2_* are the variance parameters which were estimated. Replicate data at each time point from multiple animals in different experiments were pooled and modeled simultaneously. The goodness-of-fit criteria were assessed by model convergence, visual inspection, precision of parameter estimates, objective function values such as Akaike Information Criterion (AIC), and examination of residuals.

## Results

### Simulation of MPL Pharmacokinetics

**Figure 2:**
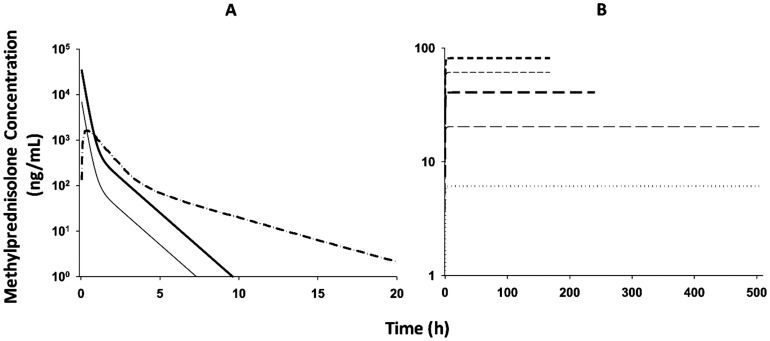
Simulated pharmacokinetics of MPL for 50/kg IM (dash-dotted line) injection, 10 mg IV (light solid line) and 50 mg IV (heavy solid line) injection groups (A) and 0.03 (dotted line), 0.1 (light long dash line), 0.2 (heavy long dash line), 0.3 (light short dash line) and 0.4 (heavy short dash line) mg/kg/h infusion groups (B). The PK parameters are listed in Table 2.

The pharmacokinetic profiles of MPL following IV drug injection or infusion and IM injection were described previously [26], [27], [33], [34], [37]. A two-compartment model with linear elimination from the central compartment was used for describing the distribution and elimination of the drug. In the case of IM dosing complex flip-flop kinetics was observed that were modeled using two independent absorption rate processes [37]. Therefore, plasma concentrations of MPL after various dosing regimens were generated using the same model and reported parameters (Fig. 2). The fixed parameters are summarized in Table 2.

### Glucocorticoid Receptor Dynamics

**Figure 3:**
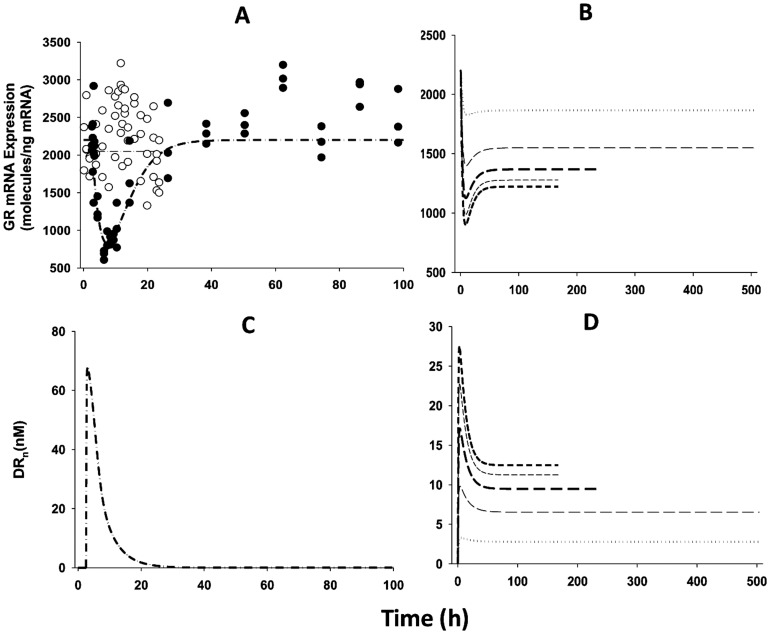
The time course of changes of GR mRNA expression (A, B) and DR_n_ (C, D) in white adipose tissue for MPL infusions (B, D), control (○), and 50 mg/kg MPL IM (•) injection (A, C). Symbols are observed data. Lines depict both the fitting results for the control (light dash-dotted line) and 50 mg/kg (heavy dash-dotted line) injection groups with Eq. (1) – (4) and simulation results for 0.03 (dotted line), 0.1 (light long dash line), 0.2 (heavy long dash line), 0.3 (light short dash line) and 0.4 (heavy short dash line) mg/kg/h infusion groups. The model parameter values are listed in Table 3.

Because receptor density and receptor mRNA data in white adipose tissue and skeletal muscle are not available for the chronic infusion study, the parameters for receptor dynamics in the single-dose studies were used and simulations were performed to predict the receptor dynamic profiles in both tissues after chronic dosing. The observed GR mRNA expression profiles along with model fittings in white adipose tissue in the control rats and the rats following IM MPL dosing are shown in Fig. 3A. In response to single MPL dosing, the GR mRNA was temporarily down-regulated with a nadir at about 10 h followed by a slow return back to the baseline. Free cytosolic GR quickly dropped to zero and recovered with two phases (data not shown). The dynamic profiles were consistent with our measurements in liver, skeletal muscle and adipose tissue [19], [24], [35], [38]. However, during chronic MPL infusion, similar to previous studies [39], [40], the receptor mRNA decreased first and then rose to a new steady-state (Fig. 3B), indicating a tolerance phenomenon. While the free cytosolic receptor stayed down-regulated during the study, the nuclear drug-receptor complex (*DR_n_*) showed an early increment followed by a decline to a new steady-state (Fig. 3D). Simulation results for the infusion study indicated tolerance phenomena for both GR mRNA and *DR_n_*. This was mainly due to GR mRNA down-regulation as there was insufficient free receptor available during continuous infusion. Because we seek to model all pooled data and accommodate different study designs in this report, modest circadian oscillations in GR mRNA expression were not incorporated into the dynamic model. By keeping the basic model structure for receptor dynamics, we obtained reasonable fittings for the GR mRNA expression in white adipose tissue (Fig. 3A) and parameter estimates similar to reported values when the circadian rhythm was included (Table 3) [24]. For liver and skeletal muscle, the GR receptor density and mRNA expression after MPL were measured and modeled previously [19], [27]. Therefore, receptor dynamic parameters in these two tissues were fixed. Sensitivity analysis indicated that the GR dynamic profiles are insensitive to changing the *k_T_* value from 58.2 h^−1^ [34], [37] to 90 h^−1^ [19], so *k_T_* was fixed to the value of 58.2 h^−1^. We previously demonstrated the apparent insensitivity of GR mRNA and GR density to higher values of *k_T_* beyond 15 h^−1^ [41]. The estimated and fixed parameters for receptor dynamics in the three target tissues are listed in Table 3.

**Table 3:** Receptor dynamic parameters of methylprednisolone.

Parameter (units)	Definition	Value	CV%
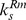 (fmol/g/h)	Synthesis rate constant for GR mRNA	0.416 ***^f^***/2.90***^g^***	fixed
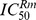 (fmol/mg protein)	DR_n_ required for 50% inhibition of GR mRNA production	15.6 ***^f^***/26.2***^g^***/0.911***^h^***	fixed
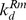 (h^−1^)	Degradation rate constant for GR mRNA	0.427 ***^f^***/0.139***^h^***	14.9 ***^b^***
*k_on_* (nM^−1^·h^−1^)	Association rate constant	0.016***^f^***/0.0033***^g^***/0.0027***^h^***	fixed
*k_re_* (h^−1^)	DR_n_ loss rate constant	1.31 ***^f^***/0.57***^g^***/0.618***^h^***	fixed
*R_f_*	Recycling factor	0.93 ***^f^***/0.49 ***^g^***/0.72***^h^***	fixed
*k_T_* (h^−1^)	Translocation rate constant	58.2 ***^f,h^***/0.63***^g^***	fixed
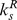 (fmol/mg protein/fmol mRNA/g/h)	Synthesis rate constant for GR	0.00196 ***^f^***/0.777***^h^***	fixed
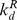 (h^−1^)	Degradation rate constant for GR	0.05 ***^f,g^***/0.035***^h^***	fixed
*R*(0) (fmol/mg protein)	GR baseline: white adipose liver skeletal muscle	77.7***^f^*** 420***^c,g^***/540.7***^b,d,g^***/328.7e,g 65.3***^h^***	13.3***^b^***
*GR_m_*(0) ***^i^*** (mol/ng RNA)	GR mRNA baseline: white adipose	2050a,f/2200***^b-e,f^***	fixed
*GR_m_*(0) (fmol/g)	GR mRNA baseline: liver skeletal muscle	18.6***^c,g^***/25.8***^b-d,g^***/3.65e,g 2.99***^h^***	fixed

a IM injection control; ***^b^*** IM 50 mg injection; ***^c^*** IV 10 mg/kg injection; ***^d^*** IV 50 mg/kg injection;

e IV infusion; ***^f^*** White adipose tissue; ***^g^*** Liver; ***^h^*** Skeletal muscle; ***^i^*** Different normalization for adipose tissue than for liver and skeletal muscle requires different units.

### Leptin and Food Intake

**Figure 4:**
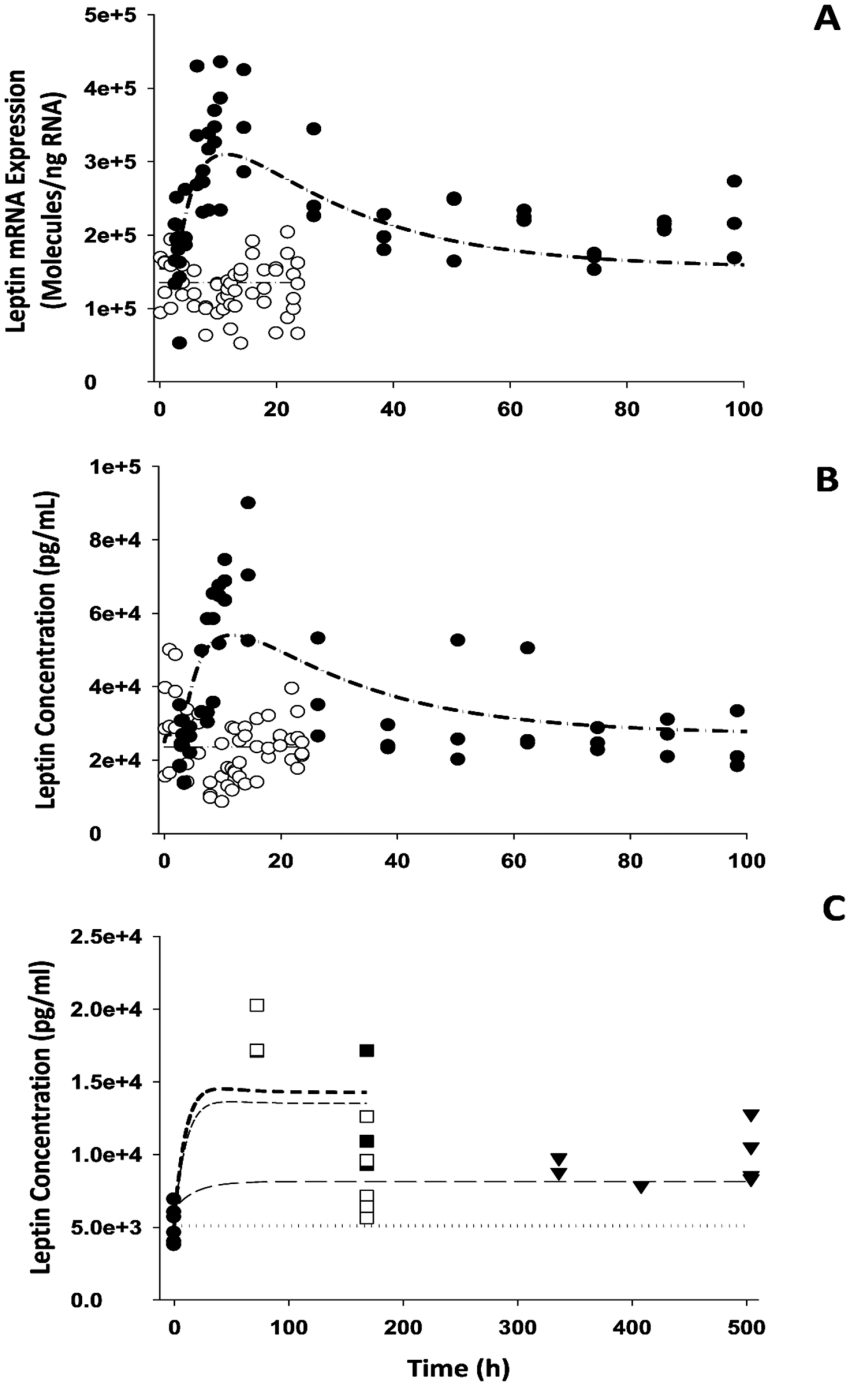
The time course of changes in leptin mRNA expression in white adipose tissue (A), plasma leptin concentrations in the circadian (B) and chronic infusion studies (C) for control (○) and 50 mg IM injection (•), and 0.03 (▾), 0.3 (▪) and 0.4 (□) mg/kg/h subcutaneous infusion groups. Symbols are observed leptin mRNA or plasma leptin concentrations. Lines are fitting results for the control (light dash-dotted line), 50 mg IM (heavy dash-dotted line) and 0.03 (dotted line), 0.3 (light short dash line) and 0.4 (heavy short dash line) mg/kg/h infusion groups. The PD parameters are listed in Table 4.

The observed leptin mRNA expression and plasma leptin concentrations along with the model fittings in the circadian-nadir study [24] and chronic infusion study [25] are shown in Fig. 4. Table 4 lists the fixed and estimated parameters for leptin dynamics. Assuming DR_n_ as the controlling factor for up-regulating leptin mRNA expression, the dynamic model (Fig. 1) well captured the changes of leptin dynamics (Fig. 4) after various MPL dosing regimens. Estimated leptin dynamic parameters are similar to previously reported values [24] except for the lower value of 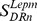
(0.0989 nM^−1^). In the circadian-nadir study, variability in leptin mRNA expression is apparent due to rich sampling within the 24 h period and circadian rhythms. Since no circadian oscillations were incorporated into the current model, stable concentrations of leptin mRNA and plasma leptin were predicted. This might also be the reason for lower estimates of 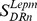
and somewhat under-predictions of peak leptin mRNA and plasma leptin as well. MPL up-regulated leptin mRNA expression as well as plasma leptin with the peak time around 12 h after drug dosing, as previously reported [24]. The chronic dosing effect of MPL on plasma leptin is shown in Fig. 4C. Compared to the profiles after acute dosing, a later up-regulation of plasma leptin was predicted followed by a slight decline to a new steady-state during the study period. The simulated profiles of *DR_n_* served as driving forces for stimulating leptin transcription. Due to the relatively low estimate of 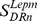, the tolerance effect of plasma leptin was only observed in the high dose groups.

**Table 4:** Pharmacodynamic parameters for leptin dynamics and food intake.

Parameter (units)	Definition	Value	CV%
_Leptin dynamics_			
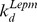 (h^−1^)	Leptin mRNA loss rate constant	0.0427	18.3
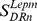 (nM^−1^)	Stimulation of leptin mRNA production	0.0989	19.4
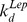 (h^−1^)	Leptin loss rate constant	4.952	244.5
*Lep_m_*(0) (mol/ng)	Baseline leptin mRNA concentration	135600 a/155100 ***^b^***/170000 ***^c-h^***	3.23^a^/4.88 ***^b^***/fixed ***^c-h^***
*Lep*(0) (pg/mL)	Baseline leptin concentration in plasma	23650 a/25000 ***^b^***/5170***^c-h^***	3.21^a^/fixed***^b-h^***
Food intake			
*k_in_Food0_* (kcal/day^−2^)	Initial food input rate	171	Fixed
*k_in_FoodSS_* (kcal/day^−2^)	Steady-state food input rate	147	Fixed
*Food_max_*(kcal/day)	Maximal food intake	57.9	9.35
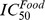 (pg/mL)	Inhibition constant of leptin on *k_in_Food_*	11550	22.4
*k_FB_* (day^−1^)	Transduction rate constant	0.0426	67.4
*k_d_* (day^−1^)	Loss rate constant for food input	1.21	Fixed
*Food* ^0^ (kcal/day)	Food intake at start of study	91.7***^c^***/80.3***^d^***/88.1***^e^***/88.5***^f^***/94.9***^g^***/88.6***^h^*** (fixed)	Fixed

a IM injection control; ***^b^*** IM 50 mg injection; ***^c^***Saline infusion; ***^d^*** 0.03 mg/kg/h; ***^e^*** 0.1 mg/kg/h; ***^f^*** 0.2 mg/kg/h; ***^g^***0.3 mg/kg/h; ***^h^***0.4 mg/kg/h.

**Figure 5:**
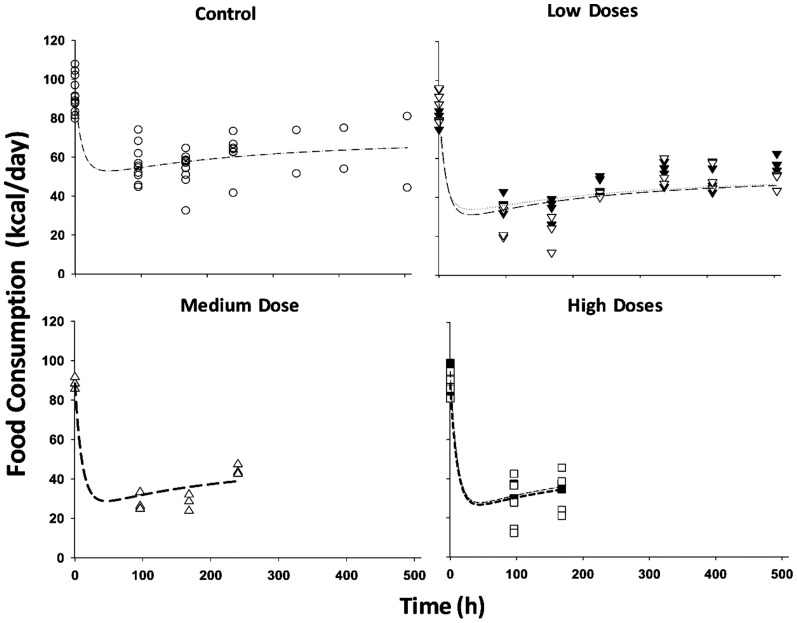
Time course of food intake (kilocalories per day) for saline (○), and 0.03 (▾), 0.1 (∇), 0.2 (Δ), 0.3 (▪) and 0.4 (□) mg/kg/h MPL infusions. Lines depict the fitting results for the food intake of saline (light dash-dotted line), 0.03 (dotted line), 0.1 (light long dash line), 0.2 (heavy long dash line), 0.3 (light short dash line) and 0.4 (heavy short dash line) mg/kg/h infusion groups. The PD parameters are listed in Table 4.

In the present study, plasma leptin served as the driving force for inhibition of food intake. In agreement with this, we observed that food consumption decreased to its nadir at about 40 h after drug dosing followed by a slow return to a new steady-state. The general model (Fig. 1) well captured the changes of food intake (Fig. 5) after chronic MPL dosing. Similar to previous modeling [25], an exponential decay function for the food input rate *k_in_Food_* was used to describe the reduction of food consumption in control rats, and the negative feedback remained the same, as we see a slow increase of food intake during drug dosing. The feedback transduction rate *k_FB_* of 0.0426 day^−1^ is similar to our previous estimate [25] and indicates a slow tolerance effect. The estimated 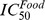
of 11550 pg/ml indicates the high potency of leptin to inhibit food consumption, which is consistent with the strong MPL effect on reduction of food intake [25]. The estimated maximal food intake (57.9 kcal/day) is in good agreement with our previous value (54.4 kcal/day) [25].

### Cyclic AMP, PEPCK and IRS-1_m_ Dynamics

The dynamic profiles of cAMP, PEPCK and IRS1_m_ were described previously [3], [19]. The parameters regulating the dynamic changes of these components were obtained from those studies and fixed to avoid over-parameterization. Table 5 lists the parameter values used in the model. After chronic dosing of MPL, tolerance phenomena were seen for all of these key elements (data not shown), which was mainly due to receptor saturation during continuous drug exposure.

**Table 5:** Pharmacodynamic parameters for cAMP, PEPCK and IRS-1_m_.

Parameter (units)	Definition	Value
Hepatic cAMP dynamics		
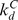 (h^−1^)	cAMP loss rate constant	0.27
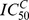 (fmol/mg protein)	Inhibition constant of DR_n_ on *k_d_^C^*	433 ***^a-f^***/17.8***^g-k^***
*cAMP* ^0^ (pmol/g liver)	Baseline cAMP concentration	806***^a-f^***/654g,h/656***^i-k^***
Hepatic PEPCK mRNA Dynamics		
*k_1_* (h^−1^)	Transduction rate constant	0.75
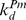 (h^−1^)	PEPCK mRNA loss rate constant	0.017
*S_s_^Pm^* (fmol/mg protein)^−1^	Stimulation of PEPCK mRNA production	2.11
*S_d_^Pm^* (fmol/mg protein)^−1^	Stimulation of PEPCK mRNA degration	2.90
*PEPCK_m_*(0) (fmol/g liver)	Baseline PEPCK mRNA concentration	430***^a-f^***/383g,h/209***^i-k^***
Hepatic PEPCK Dynamics		
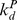 (h^−1^)	PEPCK mRNA loss rate constant	0.017
*λ*	Amplification factor	0.004
*S_max_^P^*	Maximum stimulation of PEPCK production	2.11
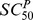 (fmol/g liver)	Stimulation constant of cAMP on *k_s_^P^*	0.0055
*PEPCK*(0)	Baseline PEPCK concentration in plasma	1.83***^a-f^***/1.4 ***^g-k^***
(µmol ATP/min/g liver)		
Muscle IL-6R1 and IRS-1		
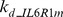 (h^−1^)	IL6R-1 mRNA loss rate constant	0.306
*S_max_IL6R1m_*	Maximum stimulation by DR_n_	3.05
*SC_50_IL6R1m_* (fmol/mg protein)	Stimulation constant by DR_n_	1.09
*k_e_* (h^−1^)	Transduction rate constant	0.144
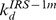 (h^−1^)	IRS-1 mRNA loss rate constant	0.313
*IC_50_DRn_* (fmol/mg protein)	Inhibition constant by DR_n_	5.95
*IC_50_IL6R1_*	Inhibition constant by IL6R-1	0.196
*N*	Number of transit compartments	5

a Saline infusion; ***^b^***0.03 mg/kg/h; ***^c^*** 0.1 mg/kg/h; ***^d^*** 0.2 mg/kg/h; ***^e^*** 0.3 mg/kg/h; ***^f^*** 0.4 mg/kg/h;

g Saline IV injection; ***^h^***10 mg/kg injection; ***^i^***50 mg/kg injection; ***^j^***Saline IM injection; ***^k^***50 mg/kg IM injection. Parameter values were fixed from Jin et al. [3] and Yao et al. [19]

### Glucose-Insulin-FFA Dynamics

**Figure 6:**
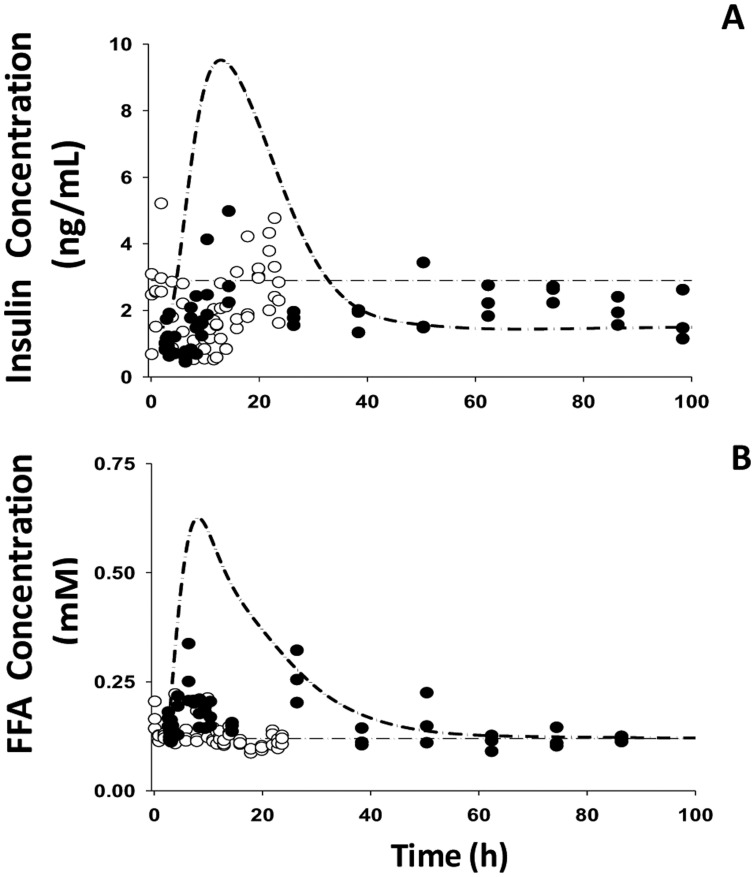
The time course of changes in plasma insulin (A) and FFA (B) for saline (○), and 50 mg/kg IM (•) MPL injections. Lines depict the simultaneous fitting results with Eq. (18) – (21) for saline (light dash-dotted line) and 50 mg/kg IM (heavy dash-dotted line) MPL injections. The PD parameters are listed in Table 6.

**Figure 7:**
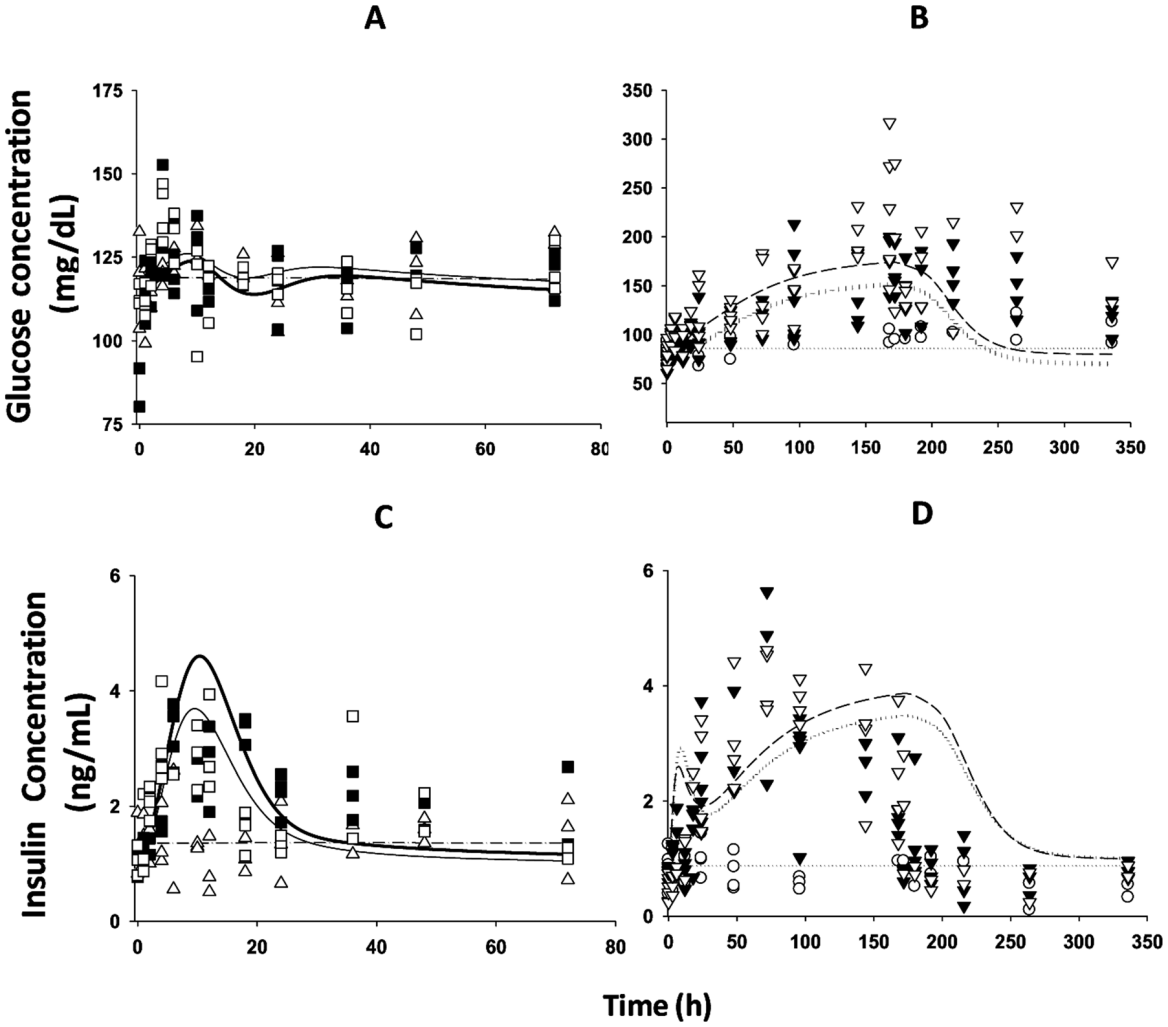
Plasma glucose (A, B) and insulin (C, D) versus time profiles during 7-day infusions (B and D) of saline (○), 0.03 (▾) and 0.1 (∇) mg/kg/h of MPL, and IV injections (A and C) of saline (Δ), 10 (▪) and 50 (□) mg/kg of MPL. Lines depict simultaneous fittings of glucose, insulin, and FFA with Eq. (18) – (21) for saline infusion (light dotted line), 0.03 mg/kg/h (heavy dotted line), 0.1 mg/kg/h (light long dash line), saline injection (light dash-dotted line), 10 mg IV (light solid line), and 50 mg IV (heavy solid line). The PD parameters are listed in Table 6.

**Figure 8:**
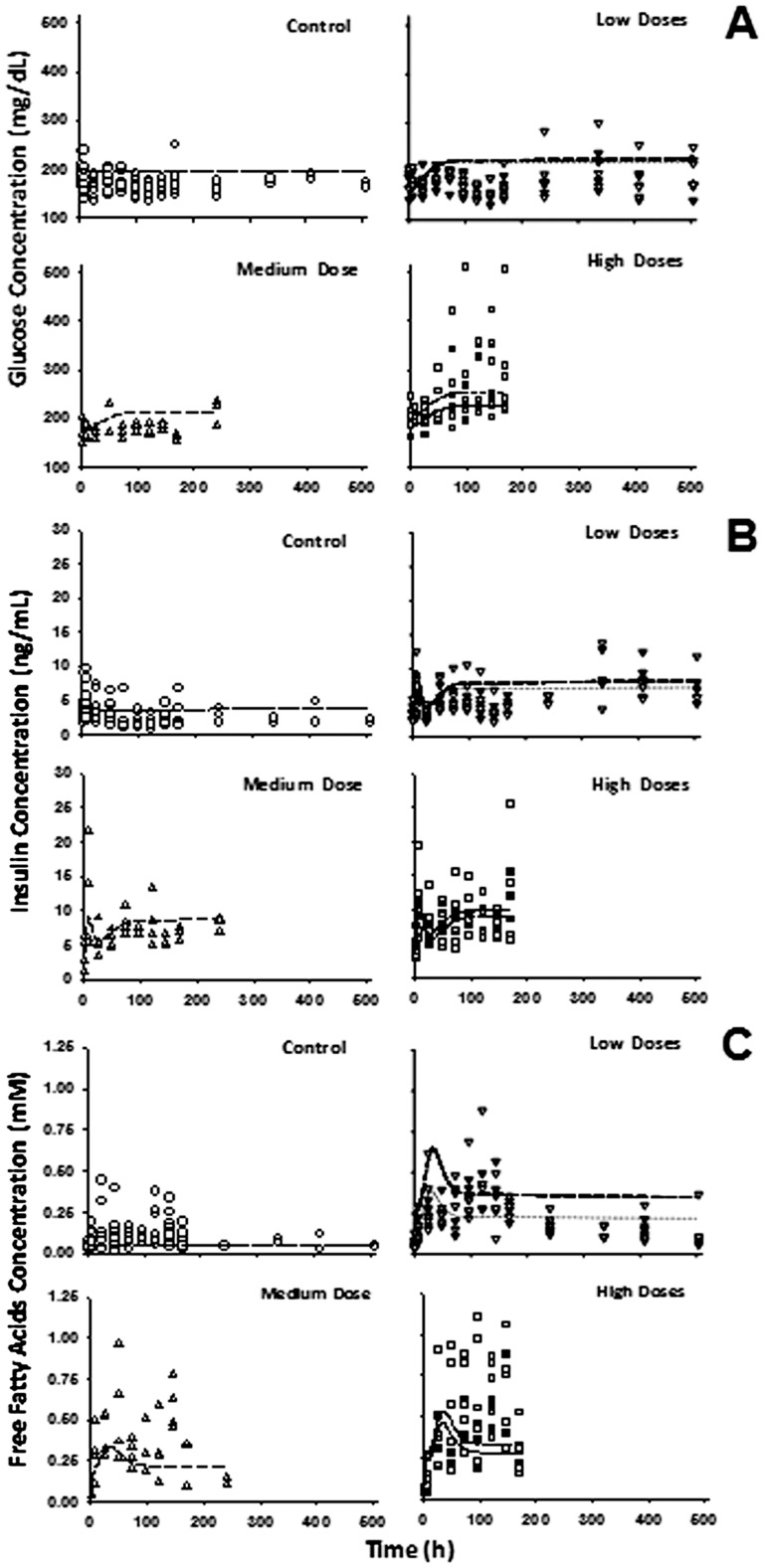
Plasma glucose (A), insulin (B) and FFA (C) versus time profiles during infusion of saline (○), 0.03 (▾) and 0.1 (∇) mg/kg/h MPL (Low doses), 0.2 (Δ) mg/kg/h MPL (Medium dose), and 0.3 (▪) and 0.4 (□) mg/kg/h MPL (High doses). Lines depict simultaneous fittings of glucose, insulin, and FFA with Eq. (18) – (21) for saline (light dotted line), 0.03 (heavy dotted line) and 0.1 mg/kg/h (light long dash line), 0.2 mg/kg/h (heavy long dash line) and 0.3 (light short dash line) and 0.4 mg/kg/h (heavy short dash line). The PD parameters are listed in Table 6.

Fig. 6–8 provide the fitting results for the circadian-nadir, chronic infusion, injection, and short infusion studies and Table 6 lists the parameter estimates. In general, our model adequately captures the time-course profiles of plasma glucose/insulin/FFA after various doses of MPL. After single doses of MPL marked changes in the concentrations of plasma insulin and FFA were observed (Fig. 6). We did not use the plasma glucose data from this acute dosing group for the modeling effort because of the strong influence of stress effects on plasma glucose caused by IM dosing as discussed by Sukumaran et al. [24]. In the chronic infusion study (Fig. 8), relatively stable plasma glucose, insulin, and FFA concentrations were observed for the control rats, indicating little contribution of food effects on overall glucose regulation. For most drug dosing groups, a slight decrease in plasma glucose followed by a gradual increase to reach a steady-state throughout the 7–21 day study period was observed. This slight decrease of glucose could be mainly explained by the initial spike of insulin at about 6 h. After the initial rapid increase during the infusion study, plasma insulin slowly increased to a new steady-state. A remarkable increase of plasma FFA was observed after various doses of MPL with a peak time about 30 h followed by a decrease to a steady-state. This tolerance effect was mainly driven by the down-regulation of GR and its mRNA. In the injection and short-term infusion studies [26], a gradual increase of glucose as well as an early peak for plasma insulin, declining thereafter followed by a gradual increase were observed (Fig. 7). Due to the 3-5-fold lower baseline insulin concentrations compared to the chronic infusion study, over-predictions of the initial insulin spike were observed. When MPL infusion ceased, plasma glucose and insulin started returning to baseline. The decline of plasma insulin was underestimated and hence the model over-predicted the decline of plasma glucose accordingly.

**Table 6:** Pharmacodynamic parameters for MPL effects on glucose/insulin/FFA regulation.

Parameter (units)	Definition	Value (CV%)
Glucose Dynamics		
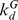 (h^−1^)	Glucose utilization rate constant	0.00521 (48.2)
*S_GI_* (mg/dL)^−1^	Glucose sensitivity	0.0305 (13.3)
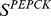 (µmol ATP/min/g liver)	Stimulation constant	0.013 (---*^h^*)
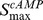	Maximal stimulation	3.45 (41.9)
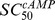 (pmol/g liver)	Stimulation constant	1.82 (55.8)
*S_Food_* (kcal/hr)^−1^	Food sensitivity	0.000346 (---*^h^*)
*λ_1_*	Power coefficient	1.55 (---*^h^*)
*G^0^* (mg/dL)	Baseline glucose	197***^a^***/184***^b^***/183***^c^/***174***^d^***/192***^e^***/211***^f^***
		119***^g^***/117***^h^***/113***^i^***/86 ***^j^***/70***^k^/*** *80* ***^l^***/190***^m,n^*** (fixed)
Insulin Dynamics		
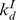 (h^−1^)	Insulin degradation rate constant	0.224 (---*^h^*)
*S_IG_* (ng/mL)^−1^	Insulin sensitivity	2.87 (53.0)
*S_IF_* (ng/mL)^−1^	Insulin sensitivity	0.315 (---*^h^*)
*I^0^* (ng/mL)	Baseline insulin	3.73***^a^***/3.45***^b^***/3.57***^c^***/3.97***^d^***/4.90***^e^***/3.89***^f^***/1.36***^g^***
		1.00***^h^***/1.10***^i^***/0.88 ***^j^***/1.00***^k^***/1.00***^l^***/2.9***^m^***/1.5***^n^*** (fixed)
FFA Dynamics		
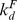 (h^−1^)	FFA degradation rate constant	0.096 (---*^h^*)
*S_FI_* (mM)^−1^	FFA sensitivity	13.3 ***^a-f, j-l^*** (31.2)/28.9 ***^g-i m-n^*** (43.9)
*k_FI_* (h^−1^)	Degradation rate constant	0.183 (---*^h^*)
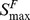	Maximal stimulation	15.0 (---*^h^*)
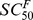 (ng/mL)	Stimulation constant	2.23 (77.3)
*F^0^* (mM)	Baseline FFA	0.053***^a^***/0.052***^b^***/0.071***^c^***/0.041***^d^***/0.053***^e^***
		0.062***^f^***/0.055***^g-l^***/0.12***^m^***/0.12***^n^*** (fixed)

Parameter values were obtained from Fang et al. [25]: ***^a^***Saline infusion; ***^b^***0.03 mg/kg/h; ***^c^*** 0.1 mg/kg/h; ***^d^*** 0.2 mg/kg/h; ***^e^*** 0.3 mg/kg/h; ***^f^*** 0.4 mg/kg/h; Jin at al. [26]: ***^g^***Saline i.v. injection; ***^h^***10 mg/kg injection; ***^i^***50 mg/kg injection; ***^j^***Saline infusion; ***^k^***0.1 mg/kg/h; ***^l^***0.3 mg/kg/h; Sukumaran et al. [24]: ***^m^***Saline IM injection; ***^n^***50 mg/kg injection; *^h^* Not estimated.

Physiological parameters 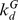, *S_GI_*,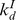, *S_IG_*, *S_IF_*, 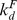, and *S_FI_* were used to describe the glucose-insulin-FFA regulation system in plasma. Different *S_IF_* values for single and chronic dosing were necessary to fit the plasma glucose/insulin/FFA profiles. A lower *S_IF_* value (13.3 mM^−1^) for single dosing was estimated compared to the value of 28.9 mM^−1^ for chronic dosing, which indicates a complex and different capacity of FFA for modulating β-cell function and insulin secretion. An empirical function *exp(−k_FI_·t)* was not used for the single dosing study as the initial spike of insulin was not observed. Instead of using plasma MPL as the driving force for GC action on FFA, the drug-receptor complex in nucleus (*DR_n_*) in adipose tissue was used. The estimate of 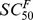
(2.23 fmol/mg protein) indicates the high potency of *DR_n_* to stimulate FFA production. In the normal glucose-insulin regulation system, increased glucose concentrations stimulate insulin secretion, and the parameter *S_GI_* of 0.0305 mg/dl^−1^ represents the glucose sensitivity, which is close to our previous estimate of 0.0178 mg/dl^−1^ [25]. However, elevated insulin stimulates glucose disposition, and a higher estimate of insulin sensitivity of 2.87 ng/ml^−1^ was fitted in our current model when both modulators were considered: the stimulation effects of FFA on insulin secretion and the enhanced peripheral insulin sensitivity effects of IRS1_m_ in skeletal muscle. This indicates that, in order to produce the same magnitude increase of glucose production, more pronounced changes in insulin are needed. Owing to the model complexity, we were not able to include the negative feedback of FFA on this stimulation effect of insulin on glucose disposition and for insulin stimulating FFA disposition. A lower value of 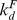 (0.096 h^−1^) compared to a previous estimate of 0.29 h^−1^ [24] was fixed according to the best fitting results. The current model yielded a lower estimate of 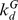 compared to our previous model which lacked multiple intermediate factors [25]. Given the low CV% associated with the parameter and rich information included in the current study, this is a more reliable estimate.

## Discussion

Glycemic control is a complex and highly integrated process where normal blood glucose is maintained by the balance between glucose production and utilization. Development of a mechanistic small systems model integrating major regulatory organs and relevant controlling factors to characterize MPL effects on glucose regulation is of importance to facilitate understanding of the complex roles and interactions of diverse factors. In developing mechanistic models of GC effects, identifying major contributing factors governing various rate-limiting processes is of primary importance. Diabetes is a multi-faceted disease involving a variety of organ systems with numerous associated biomarkers. Their inclusion and interrelationships represent challenges in model building. Our current model provides insights into GC action on glucose regulation via parallel analysis of the three most relevant target organs (liver, muscle and adipose tissue). MPL was chosen as the model drug because of its favorable PK properties and our extensive data. We have utilized well-established biomarkers and considered the importance of their contributions to glucose regulation. Earlier work demonstrated that MPL induces liver-specific cAMP and PEPCK [3] and IRS-1 in skeletal muscle [33]. In liver, PEPCK is the rate-limiting enzyme for gluconeogenesis and cAMP plays an important role in glucose regulation [42]. Skeletal muscle is a major target tissue for GC-induced insulin resistance and is responsible for about 80% of insulin-stimulated total glucose disposal. IRS-1 mRNA in skeletal muscle is down-regulated after acute and chronic MPL [19]. It is involved in signal transduction for mediating insulin effects, thus affecting glucose metabolism [43]. Furthermore, white adipose tissue is an additional target organ related to adverse metabolic effects of GC [44]. Leptin and FFA, two bioactive molecules mainly produced in adipocytes, contribute to insulin resistance and are important regulators between tissues [7]. In addition, leptin is an important adipokine responsible for energy metabolism by suppressing appetite [6]. Therefore, both serve as intermediate factors contributing to GC-induced hyperglycemia. Our selections of potential biomarkers were mainly based on currently known mechanisms and literature data; however, this does not exclude the possibility of additional relevant factors.

One important function of the liver is to maintain normal blood glucose concentrations. Glucose originates from dietary carbohydrates and via glycogenolysis and gluconeogenesis in liver. Liver is also a major site for glycogen storage via glycogenesis. In our model, parameter *S_Food_* reflects the efficiency of food intake in controlling the exogenous source of glucose input. The value of 0.000346 (kcal/h)^−1^ was fixed for *S_Food_* to a previous estimate [25], indicating that a 0.2% increase in 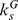
occurs when food consumption is 120 kcal/day. Furthermore, the parameters *S^PEPCK^* (0.013 µmol ATP/min/g liver), 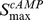
(3.45) and 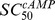
(1.82 pmol/g liver) represent the efficiency of endogenous glucose arising from hepatic glucose output. Considering the magnitude of change of PEPCK (1-2-fold after single doses and chronic infusion) and cAMP (100's for injection and 1000's for chronic infusion), hepatic glucose output represents the major pathway for glucose production and GC regulate glucose production mainly by affecting this source.

White adipose tissue is the other important target of GC [6] and serves to store excess energy as well as maintain energy homeostasis. Previous studies have shown that MPL elevated circulating plasma leptin and FFA along with up-regulated leptin mRNA expression in white adipose tissue [24], [25]. In the current study, because of limited data availability for receptor dynamics in white adipose tissue [24], parameters from single MPL dosing [24] were used to simulate the receptor dynamic profiles after chronic dosing. The *DR_n_* was used as the driving force for the transcriptional regulation of leptin expression in white adipose tissue, and our model in general well captured the profiles of leptin mRNA and plasma leptin after both single and chronic MPL (Fig. 4). However, our model under-predicted plasma leptin concentration for the first time point in the high dose group (0.4 mg/kg/h) of the chronic infusion. This may be due to insufficient data sampling as we only have few data points with appreciable variability and needed the assumption of similar GR dynamics in acute and chronic infusion studies. The misfit also indicated that the experimental data for GR mRNA expression and *DRn* in white adipose tissue of the chronic infusion study may be needed in order to validate the assumption. Based on the physiological role of leptin to suppress appetite, assuming an inhibitory effect of leptin on food input rate adequately fitted the food intake data after chronic infusion of MPL (Fig. 5). However, in comparison with hepatic glucose output, the food consumption effect on exogenous glucose input seemed to be modest. The GC directly stimulated lipolysis in adipocytes by increasing hormone-sensitive lipase and triglyceride lipase and use of antagonists for GR blocks these effects [45]. Therefore, *DR_n_* was used as the driving force to stimulate the production of FFA. During chronic infusion of MPL, our model predicts a quick increase of *DR_n_*, which peaks within 6 h followed by a relatively slow decline to a new steady-state at about 40 h. Correspondingly, plasma FFA peaks about 32 h and then returns to a new steady-state higher than the baseline, indicating a tolerance effect after chronic MPL dosing. In contrast, after single MPL doses, the model predicted an initial faster increase of *DR_n_* to a peak value at 30 min, followed by a rapid decline to baseline [24]. Plasma FFA was stimulated to peak at about 5 h after dosing and return back to baseline. Our model captured the up-regulation of FFA by MPL generally well, but overestimated the plasma FFA and the return to baseline (Fig. 6). This discrepancy might be caused by the different assay methods used which resulted in different baseline values (0.12 mM in circadian study vs 0.05 mM in chronic infusion study). We tried to factor in a scaling parameter to differentiate the assay methods used between the two studies [24], [25]; however, due to lack of relevant information, additional parameter estimates could not be obtained with reasonable precision. The antilipolytic effect of insulin was not included owing to model complexity, which may contribute to the underestimation of the rate of decline of plasma FFA in our current model.

A major target for GC-induced insulin resistance is skeletal muscle. After GC treatment, a shift in energy metabolism from glucose to FFA, impaired insulin signaling transduction, and reduced glucose uptake into skeletal muscle have been associated with insulin resistance [43], [46]. Our extensive microarray analysis provided a transcriptional basis for the development of insulin resistance in rat skeletal muscle in response to GC challenge [43]. Notably, the decline in IRS-1 in skeletal muscle may inhibit insulin-stimulated glucose uptake in type II diabetic patients and insulin-resistant animals [47], [48], [49]. Consistent with this, down-regulation of IRS-1 mRNA, a central factor in insulin signaling, was observed after GC dosing [19], [43]. In our model, the decline in IRS-1 mRNA was proposed to account for GC-induced insulin resistance in skeletal muscle, and the time course changes of *IRS1_m_*/*IRS1_m0_* was used to modify the sensitivity factor *S_IG_*. As concentrations of *IRS1_m_* decline, more insulin would be needed to account for the decrease in *S_IG_* in order to exert the same effect for stimulating glucose disposition, thus leading to the development of insulin resistance. The detailed profiles of *IRS1_m_* after single and chronic dosing of MPL were described previously [19]. After bolus MPL, the decline of *IRS1_m_* may account for the predicted glucose profile (Fig. 7A) with two different phases. During the infusion study, *IRS1_m_* decreases to lower than baseline, which may in part explain the increased plasma glucose in association with hyperinsulinemia. Due to lack of information for the temporal profile of IRS-1 proteins in skeletal muscle, IRS-1 mRNA was built into the current model. Future measurements of protein concentrations will be helpful in upgrading the current model and improving our understanding of disease progression during long-term MPL dosing.

The glucose-insulin feedback system seems to play a central role in maintaining glucose homeostasis by coordinating multiple organs, including the functions of β-cells, liver, muscle, and adipose tissue. Elevated insulin secretion from pancreatic β-cells counterbalances the increased plasma glucose with a major effect in increasing peripheral glucose utilization. In order to maintain normal plasma glucose, appropriate adaptive responses from different tissues provide the key for proper co-ordination of peripheral insulin sensitivity and insulin secretion. In response to the diabetogenic action of GC, some major systemic effects include: increased gluconeogenesis and hence greater hepatic glucose output from liver; increased protein breakdown from skeletal muscle which increases the supply of substrates for hepatic gluconeogenesis; lipolysis and differential production of adipokines from adipose tissue, and peripheral insulin resistance. Among these, appropriate insulin secretion from pancreatic β-cells seems to play a major role in compensating for GC-induced hyperglycemia. Rapid elevations of insulin were observed in response to both single and chronic MPL dosing; however, different dynamic patterns necessitated the utilization of two different sensitivity parameter values of *S_IF_*. A relatively lower value of *S_IF_* (13.3 mM^−1^) was found for single dosing in comparison with a higher estimate of 28.9 mM^−1^ for chronic dosing, indicating that the rapid increase in plasma FFA could impair the capability of insulin secretion from β-cells [50]. This difference in S_IF_ could also indicate some incompatibility in the data, possibly the different assays used for measuring insulin. As reported previously [25], an empirical function *exp(−k_FI_·t)* was needed to account for the biphasic patterns of plasma insulin during the infusion study. Some discrepancies were seen after simultaneous modeling of different data sets from chronic [25], [26] and acute studies [24]. In the single acute dose study [24], in general, our model captured the trend of up-regulation of insulin by MPL, but the initial rise of plasma insulin was over-predicted (Fig. 6). In one of the chronic studies [26] (Fig.7), the initial spike of insulin and the rate of decline of plasma glucose were overestimated and the model under-predicted the rate of insulin decline accordingly. One reason for the observed deviations could be the differences in baseline plasma insulin; additionally, previous research indicated that stress could reduce plasma insulin [51], and this might contribute to the over-prediction of the initial spike as well, since stress differences may occur among studies. Because skeletal muscle is responsible for the majority of insulin-stimulated glucose disposition and IRS-1 is a key element in regulating metabolic actions of insulin, a decline in IRS-1 transcription in skeletal muscle was used to explain the development of insulin resistance in peripheral tissue. Other mechanisms contributing to insulin resistance such as the role of FFA and decreased glucose transport were not included. These factors might account for discrepancies in model predictions.

The GC can induce insulin resistance, depending on the dose and duration of treatment [23]. As a diabetogenic hormone, it is generally thought that the risk of development or aggravating diabetes increases as GC doses increase. Consistent from our model predictions, modest changes in plasma glucose were noticed after acute MPL, even though large increases in plasma FFA and insulin were observed. Furthermore, during chronic infusion, larger increments in plasma glucose were observed. As doses increased, plasma glucose increased as well. An adaptive response of the decline in IRS-1_m_ impaired the ability of insulin to promote glucose utilization, hence leading to the development of insulin resistance. Different capacities of plasma FFA to stimulate insulin secretion under acute and chronic regimens, together with the tolerance phenomena for plasma FFA dynamics during chronic MPL infusion further exacerbated the overall system response. Therefore, successful adaptation to GC treatment resides in the proper co-regulations of peripheral insulin sensitivity and insulin secretion. In our study, quantitative modeling of the dynamic changes of some key elements in the three major target tissues not only provided a mechanistic basis to assess the inter-regulations among those entities, but also suggested important roles of different target tissues in both β-cell function and insulin sensitivity.

Various challenges were faced in developing this complex systems model. The integration of fundamental PD principles and knowledge of pharmacological and physiological processes provide the basis for development of our meta-model. The diversity of the data, the complexity of the model, the limited capability of current computer software, and need for assumptions derived from current literature make it difficult to fit all data simultaneously. Therefore a piecewise approach and partial simulations were applied. The mathematical model was developed based on our understanding of multiple cellular and tissue-level factors influencing MPL effects. Difficulty in collective fittings necessitate partitioning this integrated dynamic system into different sub-models: drug-receptor dynamics in three target tissues were fixed according to our previous results, and the *DR_n_* of each was used to model GC-driven effects on some tissue factors (liver: PEPCK and cAMP; muscle: IRS-1; fat: FFA and leptin). Parameters from previous results were fixed for analysis of the glucose-insulin-FFA dynamic system. Breaking the integrated system into parts greatly simplifies the computational process with fewer uncertainties in parameter estimates. However, incompleteness in the model components and structure might exist and the present simplification may not be optimal. The developed model was based on our existing information from pharmacology and disease pathophysiology and relied upon our accumulated, recently generated, quantitative experimental data. Nevertheless, this modeling effort provides an advancement in understanding of an important metabolic system as perturbed by a frequently employed therapeutic agent. During the model fitting process, multiple indirect mechanisms and reciprocal interactions among different components cause identifiability problems in parameter estimates. Several unidentifiable parameters had to be fixed either according to the best fitting results or previous literature values. Parameters *S^PEPCK^*, *S_IF_*, *k_FI_* and 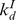
were fixed to previous values [3], [25] and *λ_1_*, 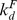 and 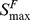
were fixed to the best model fitting results. As indicated in a recent review [52], with the growing knowledge about other substances potentially important for glucose regulation, the inclusion and selection of more key components may be necessary in future models. Lastly, further inclusions of relevant genes/proteins in different tissues are needed to fully understand drug actions at receptor/gene as well as systemic levels [19], [43], [53], [54], [55].

Some limitations of the current model exist. This report collated data from different studies. Doses, sampling times, and assay methods varied. Parameters reflecting the gene/receptor-mediated GC effects on hepatic cAMP, PEPCK mRNA, PEPCK and IRS-1 mRNA were obtained from ADX rats, and were fixed in our current model to mainly account for sources of glucose output from liver. Our system is complex with appreciable biomarker response variability and interactions, and our model is built upon extensions of previous models with inclusions of available measurements. Additional sensitivity analysis would be helpful in building confidence about the model by studying the uncertainties that are associated with each parameter. Our current report focuses on judicious use of best previous estimates of some model components (PK, receptor binding, and turnover) and joint refitting of key response measures where different study designs add improved perspectives on the behavior of the system. Not all data profiles were successfully recaptured. Such deviations are informative on how one might explore additional determinants of the system and design future studies. Because of the well-known effects of adrenalectomy on glucose regulation, including both actions of GC and adrenal medulla hormones such as catecholamines were not taken into account. Hence the resulting parameters from ADX rats might somewhat underestimate the influence of hepatic output in normal rats. Circadian oscillations in plasma glucose, insulin, and FFA were not considered. Different baseline values were used in part to differentiate the assay methods among studies and account for each study condition. In addition, the experimental procedures and animal handling could result in varied animal stress, which would contribute to different food intake profiles and glucose/insulin responses. Other physiological factors were not included such as adiponectin, which is regulated by GC [24] and relates to insulin resistance by augmenting insulin sensitivity in various target tissues [44]. Such inclusion of adiponectin caused problems with parameter estimates. However, the overall impact of other adipokines is yet to be completely understood. Other mechanistic actions of MPL omitted from the model include insulin stimulation of FFA utilization and increased FFA promoting peripheral insulin resistance.

In conclusion, a mechanistic meta-model was developed to retrospectively integrate several intensive data sets from studies in our laboratory. The model provides broader insights for various intermediate controlling processes governing MPL actions on glucose regulation. The receptor/gene/protein-mediated GC effects in liver, adipose tissue and skeletal muscle were incorporated into the model to explain physiological factors controlling various processes. Joint assessments of key elements in each target tissue suggested important roles of those entities in glucose regulation.
